# Unintended cation crossover influences CO_2_ reduction selectivity in Cu-based zero-gap electrolysers

**DOI:** 10.1038/s41467-023-37520-x

**Published:** 2023-04-12

**Authors:** Gumaa A. El-Nagar, Flora Haun, Siddharth Gupta, Sasho Stojkovikj, Matthew T. Mayer

**Affiliations:** 1 Electrochemical Conversion, Helmholtz-Zentrum Berlin für Materialien und Energie GmbH, Hahn-Meitner-Platz 1, 14109 Berlin, Germany; 2 Institut für Chemie & Biochemie, Freie Universität Berlin, 14195 Berlin, Germany

**Keywords:** Electrocatalysis, Electrocatalysis, Catalysis

## Abstract

Membrane electrode assemblies enable CO_2_ electrolysis at industrially relevant rates, yet their operational stability is often limited by formation of solid precipitates in the cathode pores, triggered by cation crossover from the anolyte due to imperfect ion exclusion by anion exchange membranes. Here we show that anolyte concentration affects the degree of cation movement through the membranes, and this substantially influences the behaviors of copper catalysts in catholyte-free CO_2_ electrolysers. Systematic variation of the anolyte (KOH or KHCO_3_) ionic strength produced a distinct switch in selectivity between either predominantly CO or C_2+_ products (mainly C_2_H_4_) which closely correlated with the quantity of alkali metal cation (K^+^) crossover, suggesting cations play a key role in C-C coupling reaction pathways even in cells without discrete liquid catholytes. Operando X-ray absorption and quasi in situ X-ray photoelectron spectroscopy revealed that the Cu surface speciation showed a strong dependence on the anolyte concentration, wherein dilute anolytes resulted in a mixture of Cu^+^ and Cu^0^ surface species, while concentrated anolytes led to exclusively Cu^0^ under similar testing conditions. These results show that even in catholyte-free cells, cation effects (including unintentional ones) significantly influence reaction pathways, important to consider in future development of catalysts and devices.

## Abstract (web summary)

Cation cross-over affects CO_2_ electroreduction process, yet the detailed effect, especially the cation effect in promising membrane electrode assemblies, needs further investigation. Here the authors reveal that unintentional cation crossover through anion exchange membranes during CO_2_ electrolysis influences product selectivity towards carbon monoxide or ethylene.

## Introduction

The utilization of CO_2_ as a carbon feedstock will be key to achieving net-zero carbon emissions and realizing a post-fossil-fuel society. Electrochemical CO_2_ reduction (CO_2_ER) into added-value commodity chemicals, powered by clean energy, offers a promising route to carbon recycling via storing renewable energy in chemical compounds^1^. CO_2_ can be electrochemically reduced into numerous products including C_1_ products (such as carbon monoxide CO, formate HCOO^−^, and methane) and C_2+_ products (including ethylene, propanol, acetate, and ethanol). Directing the selectivity of CO_2_ER to target a particular product with an adequate production rate and selectivity is necessary for its industrial application.

Gas-diffusion electrodes (GDEs) enable the direct feed of gas-phase CO_2_ to the cathode, which is important for overcoming mass-transport limitations and achieving target current densities^2–5^. The core components of a GDE-based CO_2_ER electrolyzer are the cathode, anode, and electrolyte, where the latter involves liquid solutions of dissolved salt and/or an ion-exchange membrane intended to impart selectivity to ion transport through the device. Water is typically supplied to the reactor in the form of liquid electrolytes at the cathode side of the membrane (catholyte) and/or the anode side (anolyte). High currents magnify the impact of ohmic losses across the cell, which can be significantly mitigated by employing the so-called “zero-gap” configuration wherein a catalyst-coated GDE is physically interfaced directly with the ion-conducting membrane to form a membrane-electrode assembly (MEA), without an explicit liquid catholyte channel between them^6–10^. In MEA CO_2_ER studies, it is generally observed that catalysts interfaced with cation exchange membranes are prone to significant rates of the competitive hydrogen evolution reaction at the sake of CO_2_ER, whereas cells using anion-exchange membranes (AEMs) and alkaline electrolytes are better able to suppress H_2_, making the latter favorable for this application^11,12^. This configuration (zero-gap cathode, AEM, alkaline electrolyte) has gained recent attention and resulted in demonstrated high yields and rates of C_2+_ hydrocarbon products^6,7,13^. However, much remains to be understood about processes occurring in such devices, particularly regarding dynamic behaviors at interfaces and transport of species^14^.

Although one expects AEMs to impede the transport of cations from anode to cathode in catholyte-free devices, in practice, this exclusion is imperfect, which contributes to the ubiquitous formation of solid precipitates that coincide with degradation of GDE performance due to gas flow field blockage, obstruction of the catalyst surface, and breakdown of hydrophobicity with consequent flooding^10,11^. These detrimental effects can be mitigated by strategies such as periodically rinsing the cathode with water^10,15–17^, but this does not fundamentally solve the challenge of undesired transport of co-ions (i.e., ions of the same charge polarity as membrane fixed charges, namely cations for an AEM) through the ion exchange membrane. An attractive solution would be operating MEA devices fed with pure water, but this typically results in low CO_2_ER activities, suggesting that cations are needed for carbon dioxide reduction. In a study using zero-gap Ag-based cathodes for CO_2_ conversion to CO, Endrődi et al. recently reported a strategy of intentionally infusing the zero-gap cathode with alkali cations which activates and regenerates water-fed devices, enabling high activity and promising long-term operation while mitigating degradation^18^. These observations show that understanding and controlling the behavior of cations in MEA devices is essential and deserves closer attention.

Meanwhile, numerous experimental and theoretical studies have shed light on the important roles of alkali metal cations under aqueous “H-cell” CO_2_ER conditions, in particular, their strong influence on product selectivity and activity for a variety of catalysts. Some recent review articles nicely summarize the work in this area and present a range of possible hypotheses regarding cation effects^19–22^, including suggestions that cations may contribute to buffering of local pH, stabilizing key reaction intermediates, and/or modulating local electric fields at the catalyst surface. A pair of recent studies showed that efficient CO_2_ER using acidic catholytes is possible by intentionally concentrating K^+^ ions at the cathode^23,24^, while Monteiro et al. revealed an absence of CO_2_ER activity in solutions devoid of metal cations^25^. In catholyte-free MEA-type electrolyzers, electrolyte effects have been less studied, but based on the observations mentioned above by Endrődi et al.^18^, it appears that cations play an important role despite the absence of liquid electrolytes at the cathode.

In this work, we report the observation of pronounced cation effects on zero-gap copper cathodes, namely significant and abrupt switching between CO_2_ER pathways leading either to CO or to multicarbon (C_2+_) products, influenced by merely varying the concentration of the anolyte. The effects correlate directly to the degree of K^+^ crossover through the membrane, revealing that even in catholyte-free devices, electrolyte effects have a critical influence on the selectivity of copper catalysts.

## Results

### Effect of anolyte concentration on CO_2_ reduction selectivity

**Fig. 1:**
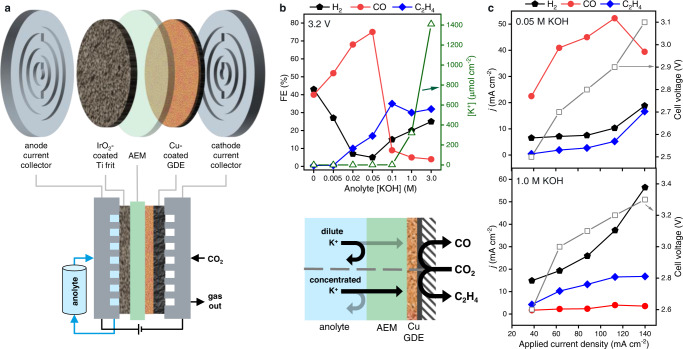
Influence of anolyte concentration on CO_2_ electrolysis selectivity. **a** Cell schematic showing the assembly of electrodes, anion-exchange membrane (AEM), and liquid anolyte. **b** Faradaic efficiency (FE) distribution of the major products H2, C_2_H_4_ and CO (complete dataset including minor products available in Supplementary Fig. 2) during operation at constant voltage (3.2 V) as a function of anolyte concentration (x-axis not to scale). Hollow triangle symbols (right y-axis) depict the amount of K^+^ extracted from the cathode flow channel, collected by injection of an H2O aliquot after 60 min continuous operation. Data were collected from separate Cu-GDE cathodes tested under each condition. The schematic below summarizes the effects of concentration on ion crossover and selectivity. **c** Measurements of partial current density (j) toward major products for Cu-GDE cells tested under constantly applied currents, ramped stepwise from low to high current, using either 0.05 M (top) or 1.0 M (bottom) KOH anolytes. Hollow squares (right y-axis) report the stabilized cell voltage at each condition.

For this study, we employed a zero-gap GDE cell based on that reported previously by Endrődi et al.^15,18,26^, here using commercial Cu nanoparticle (CuNP) catalysts spray-deposited onto commercial GDLs (see the “Methods” section for details). As summarized in Fig. 1a, the cell stack comprised a cathode current collector with radial flow pattern, Cu-coated GDE, anion-exchange membrane (PiperION), IrO_2_-coated-Ti anode, and anode current collector assembled in that sequence by compression. Humidified high-purity CO_2_ gas was fed to the cathode, while liquid anolyte from an external reservoir was pumped across the anode. No liquid catholyte was added. In CO_2_ER experiments using concentrated anolytes (KOH > 0.1 M) we observed the formation of K-containing precipitates, which contributed to performance degradation and cell failure, in agreement with previous reports^10^. Since the flux of K^+^ through the membrane is likely correlated to its original concentration in the anolyte, we postulated that the use of more dilute anolytes might be one approach to mitigate the formation of precipitates at the cathode. We found only a few previous studies which systematically varied the anolyte concentration for MEA CO_2_ER devices, mostly conducted using Ag catalysts, rarely exploring low concentrations below 0.1 M (except in pure H_2_O experiments)^18,27–29^. We therefore conducted a series of experiments in which the anolyte concentration [KOH] was systematically varied over a wide range, including concentrations <0.1 M, which normally impart prohibitive ohmic resistance when using catholyte-containing cells, but are uniquely enabled via the zero-gap configuration.

For each experiment, a pristine Cu-GDE was prepared and assembled into the cell. Each was tested using flows of KOH anolyte of different concentrations, while gaseous products were measured by online gas chromatography. A two-electrode configuration was used since, in the absence of a catholyte, it is difficult to incorporate a reference electrode for controlling or measuring the cathode potential. Thus, the voltages reported here correspond to bias between the anode and cathode current collectors. In the first experiments, a fixed bias was applied to the cell (*vide infra* for constant-current experiments). Other than the anolyte concentration, all other system parameters were kept constant.

Fig. 1b depicts the measured faradaic efficiency (FE) toward major products as a function of anolyte concentration, as determined by repeated gas chromatograph (GC) injections. At higher values (≥0.1 M), typical behavior for Cu is observed – C_2_H_4_ is the dominant product along with the H_2_ evolution side-reaction, and CO formation is minimal. A variety of other products are also detected but omitted here for clarity (see Supplementary Fig. 2 for complete characterization). In contrast, using [anolyte] <0.1 M resulted in a drastic change in selectivity – CO became the dominant CO_2_ER product while C_2_H_4_ was suppressed. At 0.05 M KOH, CO peaked at over 75%, while H_2_ was under 5%. Interestingly, the selectivity and formation rate of C_2_H_4_ decreased progressively with decreased anolyte concentrations. Even using pure water without any (intentional) alkali metal cations resulted in the selective formation of CO, albeit accompanied by significant H_2_.

Additional experiments were conducted to evaluate the possible influence of voltage- vs. current-controlled operation, electrolyte type, and membrane type on the observed behavior. Comparisons based on rate-controlled experiments enable us to normalize the kinetics of cell operation. We conducted controlled-current experiments using 0.05 M and 1.0 M KOH anolytes (conditions favoring CO and C_2_H_4_, respectively) using single Cu-GDE cathodes for each electrolyte condition, stepping progressively from low to high current while measuring products. As shown in Fig. 1c, when varying the current density across the range 38–140 mA cm^−2^, CO persisted as the dominant product in the 0.05 M KOH cell, while with 1.0 M KOH the major products were C_2_H_4_ and H_2_. In the latter case, the CO yield remained very low across all currents tested, even at the lowest currents, which correspond to a low bias voltage, which is an important observation since it shows that the observed CO/C_2_H_4_ selectivity switch is not arising simply due to differences in applied potential at the cathode.

To examine possible electrolyte and pH effects, we repeated the experiment of Fig. 1b using KHCO_3_ solutions as anolyte. Similar CO_2_ER selectivity trends as a function of anolyte concentration were observed (Supplementary Fig. 3a), suggesting that K^+^ concentration is a key factor influencing selectivity, despite different anion species and pH. It must be noted that when using recirculating alkaline electrolytes, equilibration of dissolved CO_2_ gradually converts the solution into CO_3_^2−^/HCO_3_^−^ form, with a concomitant decrease in pH (Supplementary Fig. 2b)^30,31^. Hence, experiments starting with KOH or KHCO_3_ eventually reach similar electrolyte conditions.

As an initial effort to assess the generality of our findings among membrane types, we repeated the study of anolyte concentration dependence using Fumasep FAA-3 membranes. As shown in Supplementary Fig. S4, a trend very similar to that of Fig. 1b was observed, with the major product switching progressively between C_2_H_4_, CO, and H_2_ as the anolyte ionic concentration varied from high to low. Important to note is that when tested at the same cell voltage, the Fumasep cell generated much smaller current densities compared to the PiperION case, likely due to increased resistance. This provides further validation that the observed selectivity trends are not simply due to differences in cell kinetics.

The observations described above suggest that the CO/C_2_H_4_ product selectivity trend is governed primarily by the anolyte concentration, even for varied anolyte type (KOH/KHCO_3_), operation mode (voltage or current control), or AEM type.

### Quantification of K^+^ crossover

In the experiment of Fig. 1b, the only parameter that varied was the concentration of KOH used as anolyte, which we hypothesized could influence the flux of K^+^ across the membrane. To assess the movement of cations, we performed in situ rinsing of the GDE to collect and quantify K^+^ reaching the GDE backside. For each [KOH] experiment in Fig. 1b, after continuous CO_2_ER operation for 60 min. an aliquot of pure water was injected into the gas stream, carried by the CO_2_ through the cathode gas flow channel and out of the cell, and collected in a downstream trap for subsequent quantification by ICP-OES. This approach allows us to assess the relative content of soluble K^+^ accessible at the gas diffusion side of the electrode following extended operation under different conditions. The moles of collected K^+^ per electrode surface area are shown in Fig. 1b (right y-axis), revealing a clear trend that coincides with the inflection of the selectivity—for [KOH] <0.1 M, the detected K^+^ is low and varies only gradually with [KOH], whereas there is a steep increase across higher concentrations reaching significant magnitudes of K^+^. This result suggests that the amount of K^+^ reaching the cathode is likely a determining factor in the observed selectivity trends.

Further experiments focused on the anolyte conditions favoring CO (0.05 M) and C_2_H_4_ (1.0 M). To look for changes in K^+^ levels over time, the rinsing procedure was repeated periodically on cells operating continuously (Supplementary Fig. 5). The result shows that dilute anolyte maintains low levels of K^+^ at the cathode, whereas using concentrated anolyte results in ca. two orders of magnitude greater amounts. Cation quantification was also conducted during controlled-current testing, resulting in comparable trends (Supplementary Fig. 6), indicating that across a range of currents and voltages, the concentration of the anolyte and resulting flux of K^+^ to the cathode appear to be the key determining parameters influencing the selectivity switch. The general trend is summarized schematically in Fig. 1b.

### Reversibility and continuous operation

**Fig. 2:**
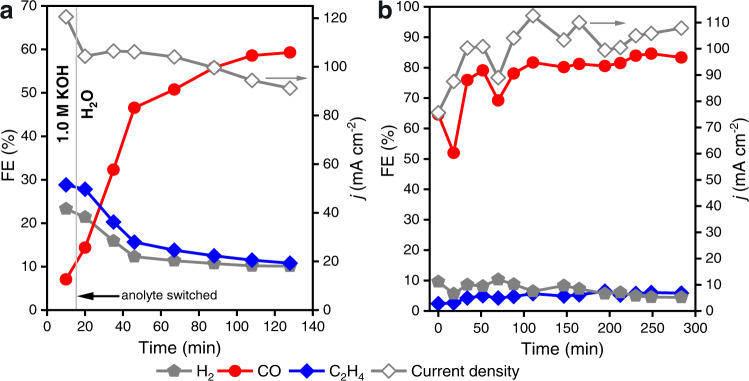
Reversibility and stability of cation effects under continuous operation. **a** FE and current density vs time for a device tested continuously with initial anolyte 1.0 M KOH, which at 10 min was exchanged with pure water. **b** FE and current density vs time for a cell with 0.05 M KOH anolyte operated continuously. All cells used an IrO_2_ anode and were tested under a two-electrode bias of 3.2 V.

We next wondered whether the observed effects of K^+^ on the cathode reactivity are reversible, *i.e*., whether the selectivity trends will switch when the electrolyte is exchanged. If water-solvated cations which crossed to the cathode are responsible for the selectivity trends, then the activity may be switchable by changing the electrolyte. To examine this, we conducted an experiment in which the cell was first operated with 1.0 M KOH anolyte for 10 min. followed by exchanging the anolyte with pure H_2_O for continued operation. Fig. 2a shows the resulting product selectivity over time, where the starting behavior correlated with the 1.0 M case of Fig. 1b (primarily C_2_H_4_ and H_2_ with minimal CO), then gradually but significantly transformed toward CO production following the switch to H_2_O anolyte. This result suggests the cation effect is indeed reversible, and that K^+^ can diffuse away from the cathode when the concentration gradient is reversed. This implies that a dynamic equilibrium between anolyte, membrane, and cathode impacts the distribution of cations across the cell, and that cations are not continuously driven to the cathode by the electric field or as primary charge-carrying species. Note that although the KOH solution was exchanged with pure H_2_O in the anolyte reservoir, residual K^+^ persists in the system, which likely contributes to the long-term behavior of the device, producing mainly CO with keeping both H_2_ and C_2_H_4_ below 10%.

The configuration yielding optimal CO selectivity (0.05 M KOH) was tested under constant bias for a period of continuous operation at 3.2 V (Fig. 2b). After an initial stabilization period, the CO FE reached 80% and remained stable over the course of 5 h, while the yield of C_2_H_4_, H_2_, and all other gaseous products remains low, accounting for less than 10% FE. Liquid products were quantified after the experiment and found to be negligible. Similarly, continuous operation of a cell using 0.05 M KHCO_3_ anolyte showed stable CO generation and only low C_2_H_4_ and H_2_ formation over a several-hour test (Supplementary Fig. 3b). The stable product selectivity and current density during continuous operation suggest that this alkali cation-deficient configuration can operate under equilibrium where the cathode microenvironment favors CO production, without flooding, precipitation, or loss of conductivity, at least on the timescales tested here.

### Origin of cation transport dependence on concentration

Our experiments indicate that the observed switch in selectivity between CO and C_2_H_4_ is correlated with the degree of K^+^ crossing the membrane to interact with the MEA cathode. We consider this an important observation for the research community working on Cu-MEA devices for electrochemical conversion of CO_2_ and CO. Alkali cation effects are well known to influence copper’s CO_2_ER selectivity, and here we show that this also affects catholyte-free devices. And while many past studies have observed evidence of cation crossover through AEMs during operation in the form of solid precipitates, exactly how and why the membranes allow such significant flux of co-ions (which are expected to be excluded in ion exchange membranes) is seldom discussed. Species transport across membranes is subject to a complex interplay of parameters, including concentration gradients, electrochemical potentials, current densities, water uptake, membrane morphology, and other details^8,11,32,33^. Our results point to electrolyte concentration as a key factor influencing co-ion crossover, which can be explained by considering the Donnan exclusion effect^34–37^. As a general explanation, Donnan exclusion relates how the capacity of an ion exchange membrane to exclude co-ions from an electrolyte solution depends on the relative density of charges between the two phases – for the membrane, the activity of fixed-charge groups (i.e., ion exchange capacity); for the electrolyte, the activity of dissolved salt (i.e., ionic strength). When the electrolyte concentration is comparable to the membrane’s fixed-charge density, minimal Donnan exclusion exists, resulting in poor permselectivity based on charge type^35^. The PiperION AEM used here has an effectively fixed-charge density of approximately 2 mol dm^−3 ^^38,39^, and we observed that K^+^ readily reaches the cathode when using anolyte concentrations in this regime (Fig. 1b). Decreasing concentrations result in a progressively decreased crossover, with strong exclusion occurring for concentrations ≤0.05 M. In Supplementary Note 1, we present further discussion of the relationship between electrolyte concentration, membrane charge density, and co-ion uptake as predicted by Donnan equilibrium. We believe that these points deserve deeper attention in the electrosynthesis community, and we refer interested readers to some key literature^32–37,40,41^. A key conclusion from this analysis is that under conditions combining typical AEMs^11^ with electrolyte concentrations commonly used in CO_2_ER (≥0.5 M)^5^, significant cation exclusion should generally not be expected.

### Operando evolution of Cu using different anolyte concentrations

Selectivity trends for Cu-based catalysts are often correlated to surface speciation (e.g., Cu oxidation states) and/or morphology, as well as how these factors may evolve during CO_2_ER^42^. Although in our experiments above, every electrode was prepared identically, it is possible that the use of different anolytes affects the resulting active form of the Cu catalysts. Thus, we conducted in situ x-ray absorption spectroscopy (XAS) and glovebox-assisted x-ray photoelectron spectroscopy (quasi in situ XPS), in addition to pre- and post-electrolysis SEM and EDX to assess both aspects as a function of employed anolyte.

**Fig. 3:**
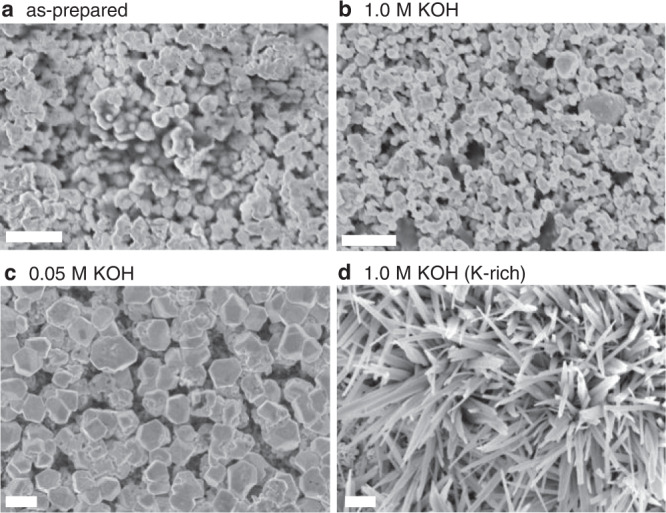
Cathode morphology and structure. SEM images of the Cu cathode surface before (**a**) and after testing in zero-gap electrolyzers under 3.2 V bias using anolytes of **b** 1.0 M KOH and **c** 0.05 M KOH. **d** Area rich in K-rich structures occurring on the part of the surface tested using 1.0 M KOH. Scale bars: 1 µm. Supplementary Fig. 6 provides cross-section images with elemental mapping.

Fig. 3 shows SEM images of the as-prepared Cu catalyst layer in comparison with samples after CO_2_ER testing using different anolytes. Supplementary Figs. 7 and 8 provide additional cross-section and element mapping images. We observed that Cu tested with 1.0 M KOH anolyte showed microstructure (grain size and porosity) that resembled the as-prepared state, without significant morphological differences (Fig. 3b). More significant changes were observed for the cathodes operated with 0.05 M KOH anolyte (Fig. 3c), which exhibited a significant increase in average grain size. Additionally, the cathodes operated with concentrated anolytes (≥0.1 M) exhibited areas of K-containing wire-like precipitates (Fig. 3d), as indicated by their elemental mapping analysis (Supplementary Figs. 7 and 8). These analyses show that the use of different anolytes influenced the structure and morphology of the zero-gap cathodes, despite the catholyte-free configuration and AEM separating the cathode from the anolyte.

**Fig. 4:**
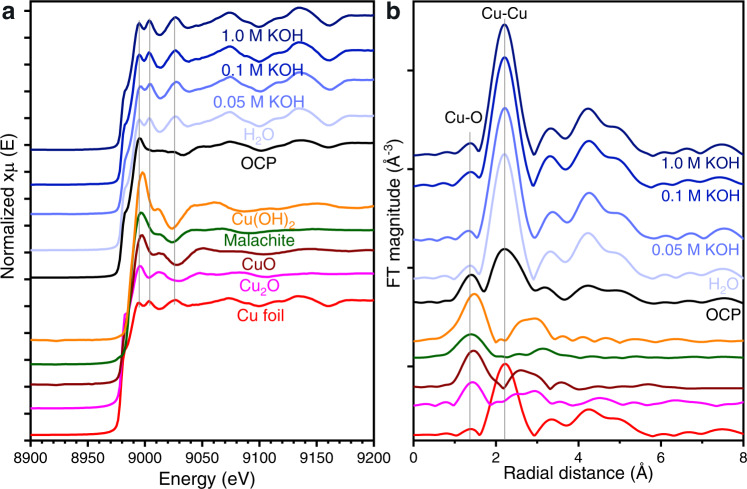
In situ X-ray absorption spectroscopy. **a** in situ Cu K-edge XANES spectra of zero-gap Cu cathodes operated with pure water and different KOH anolyte concentrations (0.05, 0.1, 1.0 M) under CO2ER conditions at 3.2 V, and **b** their respective k^2^-weighted FT-EXAFS in the k range between 3–14. Measured reference materials are shown in the bottom half.

To examine the catalyst under operating conditions, we conducted in situ XAS to track the catalyst and its chemical composition during CO_2_ER. As shown in Fig. 4, Cu K-edge XAS measured under 3.2 V operation resulted in spectra that closely resemble metallic Cu, regardless the anolyte concentration used, suggesting the complete reduction of the bulk material (within the limit of detection of this bulk-sensitive method). Notable here was that the zero-gap configuration allowed the design of an in situ cell which had the same geometry as the laboratory cell used for CO_2_ER studies, which we validated under CO_2_ER operating conditions to give comparable current density and product selectivity (Supplementary Fig. 9), meaning such a cell can be used for study under true operando conditions.

**Fig. 5:**
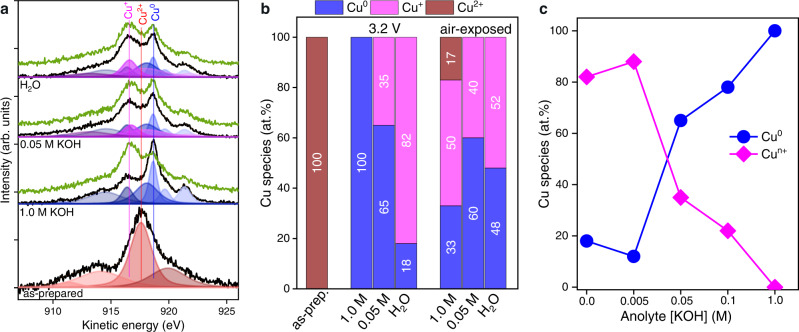
XPS analysis of surface speciation. **a** Cu LMM Auger spectra of as-prepared sample and samples measured at 3.2 V using different anolyte concentrations (H_2_O, 0.05 M and 1.0 M KOH). Black lines are raw data obtained without air exposure, green lines are re-measurements following ca. 30 min. air exposure, and shaded curves result from fitting to the indicated Cu species (orange dotted lines are fitted spectra). **b** Resulting quantification (atom %) of the Cu speciation derived from Auger spectra fitting. **c** The metallic and total oxidized Cu fractions plotted against the anolyte condition.

While hard X-ray XAS allowed operando study, the signals are bulk-dominated and therefore inadequate for gaining precise information about the catalytic interface. A complimentary surface-sensitive technique, so-called “quasi in situ” XPS^43,44^, was used for better evaluating the surface speciation as a function of employed anolyte. Here, cells are subjected to CO_2_ER inside an inert O_2_-free glovebox, followed by disassembly and transfer of the Cu-GDE to the XPS analysis chamber under vacuum using a gastight transfer arm. This approach avoids sample oxidation in air, which can be significant for Cu as stated below. The results are summarized in Fig. 5 and Supplementary Fig. 10. The Cu Auger region (Fig. 5a) was used for deconvoluting and quantifying copper speciation^45^. The surfaces of the as-prepared Cu cathodes (exposed to air) are exclusively composed of CuO species (Fig. 5b), as expected for Cu metal with a native oxide layer. For the Cu cathodes operated at 3.2 V and flowing 1.0 M KOH anolyte (C_2+_ products-selective), the oxide features disappeared entirely and the surface appeared fully reduced, while ones tested using either pure H_2_O or dilute anolyte (CO-selective cathodes) exhibited a mixture of Cu_2_O (35–45 at.%) and metallic Cu (65–55 at.%) surface species. As a general trend, we found that the surface metallic Cu species increases, while oxidized species decrease, along with increasing anolyte concentration (Fig. 5c). This highlights a possible role of the crossed-over cations on the cathode surface speciation. Moreover, the Cu cathodes operated with dilute anolyte exhibited much higher Cu_2_O surface species (even after days of air exposure) compared to operated cathodes with 1.0 M KOH.

## Discussion

Previous studies of CO_2_ER MEAs commonly observe K^+^-containing precipitates under certain operating conditions, and models have been developed to predict threshold current densities at which the carbonate generation rate brings the K_2_CO_3_ concentration beyond the solubility limit^8,10^. But the specific impacts of unintended alkali metal cation crossover on the CO_2_ER reactions, and the factors affecting the magnitude of the crossover, have received less attention in studies of Cu-based zero-gap cathodes. Meanwhile, the significant impact of cations on CO_2_ER at Cu electrodes has been extensively studied in H-cell configuration experiments and by computational methods^19–21,46^. Our observations show the importance of linking these concepts and evaluating their impacts using practical device configurations.

The selectivity switching between CO and C_2_H_4_ as a function of K^+^ reaching the cathode may be interpreted in the context of existing hypotheses about CO_2_ER cation effects. Overall, three main hypotheses have been debated in the literature to explain cation effects: (i) stabilizing the reaction key intermediates via electrostatic interactions, (ii) regulating the local CO_2_ concentration by buffering the interfacial pH and (iii) tuning the local electric field^19–22,46^. An experimental-theoretical study by Resasco et al. highlighted the essential role of cations in facilitating the C-C coupling step, and hence enhancing the selectivity towards C_2+_ products^47^. They reported higher C_2+_ production rates in the presence of heavier alkali cations attributing to their higher concentration (due to a more compact hydrated radius) compared to the lighter ones in the outer Helmholtz plane. In a related direction, Huang et al. reported that the enrichment of cation concentration near the electrochemically active sites facilitates CO_2_ activation and C-C coupling in an acidic medium^24^. They also observed H_2_ selectivity to scale inversely with [K^+^], similar to our observations across a range of low anolyte concentrations. Conversely, at higher concentrations, we see H_2_ begin to climb with [K^+^]. In studies on Au in alkaline media, Goyal et al. observed an increase of hydrogen evolution with the increase of the near-surface cation concentration, deducing that high cation coverage enhances the rate of the sluggish Volmer step^48^. Hence our observed effects of K^+^ on CO vs C_2_ selectivity, as well as H_2_ evolution, generally follow trends demonstrated in aqueous H-cell studies.

A striking exception is our observation of a range of conditions in which CO forms as a major product of copper. In previous studies of aqueous CO_2_ER cation effects on Cu, alkali metal cations were found to mainly influence the activity toward C_2+_ products relative to H_2_ evolution, with only minor yields of CO regardless of the applied potential or type of alkali metal cation^42,47,49,50^. This discrepancy may arise from the different range of cation concentrations attainable in the different cell configurations. In aqueous bicarbonate solutions, it is challenging to systematically study low concentrations due to the significant ohmic resistances across dilute electrolytes, which would require prohibitively large cell voltages in H-cells (or catholyte GDE cells)^8^. Since the zero-gap configuration is less sensitive to electrolyte ohmic losses, it provides a unique opportunity to operate cells with very dilute electrolytes and thereby limit the cations reaching the cathode interface via the mechanisms of membrane ion exclusion. Our observation of predominantly CO under cation-deficient conditions may provide evidence for a coverage- or concentration-dependent mechanism in which the C_2+_ pathway is enabled only at sufficiently high cation levels at or near the interface.

This study identifies that under certain conditions, even pure Cu catalysts can exhibit selective CO production. This could present an interesting avenue to develop Cu-based CO_2_-to-CO electrolyzers as an alternative to more complex bimetallic compositions^51,52^ or rare metals typically regarded as the best CO-selective catalysts (i.e., Ag, Au). However, the CO production rate and efficiency reported herein are inferior compared to the state of the art, so further investigation and optimization would be needed to assess whether this approach could be suitable for practical CO production.

As the tested anolyte concentrations approached zero, the CO_2_ER activity progressively decreased (Fig. 1b), suggesting that some cations are necessary even for the CO pathway (note that even in our 0 M anolyte experiments, some residual K^+^ is still present due to activation of the membranes in KOH solutions.). We compare this observation with the study of Monteiro et al., who used acidic electrolytes as an approach to attain solutions with low (or no) alkali cations while maintaining electrolyte conductivity^25^. They found CO_2_ER is sensitive to the concentration of cations even at very low levels, and made the key observation that no CO_2_ conversion is detected in the absence of metal cations in solution. Despite the use of very different cell and electrode configurations, our observations show general agreement with theirs in terms of the key role of cations in enabling CO_2_ reactivity.

Some recent studies identified cation effects on MEA reactivity using Ag-based catalysts for CO_2_ conversion to CO. Romiluyi et al. studied Ag MEAs with varied CsHCO_3_ anolyte concentration in the range 0.1–1.0 M, acknowledging that Donnan exclusion can be overcome with increasing concentration, and observing some anolyte-dependent sensitivity in the selectivity and partial current densities toward CO and H_2_ which they attributed to the presence of hydrated Cs^+ ^^27^. Endrődi identified the importance of alkali cations on the activity of Ag toward CO production and developed an approach to intentionally dose cation solutions into the GDE in order to activate and regenerate its activity^18^. Their results, combined with our current observations and the above-mentioned studies conducted in acidic media, all point to the importance of alkali cations in the catalyst environment for activating CO_2_ER pathways on MEAs.

Finally, we consider possible explanations for the Cu surface speciation trends we observed by XPS as a function of anolyte concentration. Although bulk copper oxides are readily reduced during CO_2_ER under all anolyte conditions (as evidenced by operando XAS analysis), the surface-sensitive XPS showed significant differences in Cu oxidation states after the reaction. If this were simply a result of the Cu surface being exposed to different pH environments at open circuit after testing, one would expect the most alkaline electrolytes to yield the most oxidized Cu (based on Pourbaix trends). In fact, we observe the opposite—as shown in Fig. 5c, concentrated anolyte resulted in more reduced Cu, while more dilute anolytes yielded more oxidized Cu. One possibility is that local cations influence the interfacial pH at the catalyst, wherein hydrolysis of hydrated cations provides a buffering effect on local pH^48,50^. In that case, an environment with higher K^+^ concentration would produce a stronger buffering effect, keeping the local pH lower and thereby preserving metallic Cu, in comparison to dilute solutions with weaker buffer capacity, which could allow pH rise and Cu oxidation. This could also influence the observed variation in surface morphology. Furthermore, it has been shown that alkali metal cations can affect morphology evolution due to cathodic corrosion via preferential interactions with different facets^53^. It is also possible that the differences in operating current or cathode potential contributed to our observed speciation and morphology differences, and future experiments integrating a reference electrode can help deconvolute these factors. However, under the conditions of low electrolyte concentration studied here, high electrolyte resistances precluded the use of reference electrodes placed in the anode compartment.

This study reveals the major impacts of the unintended cation crossover from the anolyte on the performance of zero-gap Cu cathodes for electrochemical CO_2_ reduction. The anolyte concentration is found to be a key parameter affecting the product selectivity, despite using a catholyte-free configuration and anion-exchange membrane. Operating with dilute anolytes directs the selectivity towards CO, while using concentrated anolytes shifts the selectivity towards C_2+_ products, showing the importance of alkali metal cations in C-C bond formation pathways. The cathode surface speciation showed variation in correlation with the different cation concentrations, where the percentage of oxidized Cu species decreased with the increase of cation crossover. Further studies are needed to fully understand the role of the membrane in CO_2_ER^11^, including investigation of the influence of membrane properties (ion exchange capacity, water uptake, thickness) and operational parameters (concentration, cell potential) on important phenomena like cation crossover. In situ studies of cation movement and catalyst speciation as functions of these parameters will be valuable for understanding and optimizing CO_2_ER devices^54^. Additional insight will come from combining experimental results with modeling studies based on microkinetics and density functional theory to evaluate the impacts of, e.g., varied cation coverage at the catalyst surface and how it influences reaction steps which dictate product selectivity.

## Methods

### Electrode preparation

For cathode preparation, catalyst ink was prepared by dispersing 10 mg/ml commercial Cu nanoparticles (*d*_avg_ ~50 nm, Sigma-Aldrich) with 6.0 wt.% polytetrafluorethylene (PTFE, Sigma-Aldrich) and 0.75 wt.% ionomer (Sustainion XA-9, Dioxide Materials) in a 1:1 isopropanol/water mixture. This dispersion was spray-coated onto 8.0 cm^2^ gas-diffusion layer (GDL, Freudenberg H23C6, 250 µm) resting on a hot plate set at 185 °C, using an ultrasonic atomizer nozzle (130 kHz, Sonaer Inc.). The catalyst loading was fixed to 1.0 ± 0.1 mg/cm^2^ for all measurements.

Anode electrodes were prepared by spraying 1.0 ± 0.1 mg/cm^2^ of high surface area Ir black nanoparticles (*d*_avg_ ~5 nm, Fuel Cell Store) using a hand-held airbrush onto 8.0 cm^2^ porous Ti frits (Changsheng Titanium Co., Ltd, 1.0 mm thickness and 225 microns average grain size) on a 185 °C hot plate. The catalyst ink was first prepared by mixing 19 mg/ml Ir black and 15 wt.% Sustainion ionomer in 1:1 water/isopropanol mixture. Prior to coating, the catalyst inks of both anode and cathode were homogenized in an ultrasonic bath for 20 minutes.

PiperION anion-exchange membranes (20 µm thickness, Fuel Cell Store) were used to separate the anode and cathode. The membranes were received in the bicarbonate form, and before use were converted into OH^-^ form by soaking in 0.5 M KOH solution for 2.0 h at room temperature, replacing with fresh KOH solution after 1 h. The membranes were then rinsed thoroughly with milli-Q water, and stored in a fresh 0.5 M KOH for at least 24 h before use.

### Electrochemical testing protocols

The testing system configuration is summarized in Supplementary Fig. 1. A catholyte-free electrolyzer reactor was used for all experiments herein (eChemicles Zrt., CO_2_ electrolyzer test cell), based on the designs reported in the publications of Endrődi et al.^15,18,26^. The gas-diffusion electrode (GDE) loaded with Cu catalyst was placed atop the cathode current collector, held in place by a 200-µm-thick PFTE spacer gasket to set the compression ratio at 15%-20% in relation to the GDE thickness. In the anode compartment, Ti frit electrodes were oriented with their Ir-coated faces contacting the anion-exchange membrane. The electrolyzer elements were assembled directly on top of each other starting from the anode side and were held together by six bolts tightened to 3 Nm torque. The electrolyzer has 8.0 cm^2^ of active electrode surface area. During CO_2_ electrolysis, humidified CO_2_ gas was continuously flowed to the backside of the gas-diffusion cathode at a constant rate of 60 ml/min using a mass flow controller. The humidification was achieved by bubbling the gas flow through 15 ml milli-Q water in a gastight 25 ml DURAN bottle. At the anode side, 25 ml of aqueous electrolyte solution (KOH or KHCO_3_) of various concentrations were recirculated in the anode compartment at a constant flow rate of 21 ml/min using a peristaltic pump and an external flask as a reservoir. All electrochemical measurements and components were operated at ambient temperature without specific thermal control.

The electrochemical measurements were carried out using a Biologic SP-240 potentiostat equipped with a 4 A current booster in a two-electrode configuration. The total cell voltage is defined as the voltage difference between the anode and cathode. No *iR* correction was used. EIS spectra were collected at an open circuit and at a constant voltage (2.5 V) before and after the CO_2_ electrolysis across the frequency range of 100 kHz – 1.0 Hz.

The composition of the gaseous products in the cathode gas outlet stream was analyzed using in-line gas chromatography (SRI GC, MG#5) every 20 min. This GC was equipped with a thermal conductivity detector (TCD) for H_2_ detection, and flame ionization detector (FID) with methanizer for CO and hydrocarbons quantification. The quantification of non-volatile liquid CO_2_ER products (formate and acetate) was conducted using Ultra-High-Performance liquid chromatograph (Thermo Scientific UltiMate 3000 series) with UV variable wavelength (UltiMate 3000, Dionex) and refraction index (RefractoMax 520, ERC) detectors, and a HyperREZ XP H+ column, using a mobile phase of 5 mM H_2_SO_4_(aq). The quantification of volatile liquid CO_2_ER products (including methanol, ethanol, and propanol) was performed using gas chromatograph (GC, Thermo Scientific, Trace 1310) via a heated headspace autosampler and FID detector. Liquid samples from the recirculated anolyte solutions, and the solution captured via downstream 10 ml cold liquid water trap from the cathode side during CO_2_ER, were analyzed for quantification of liquid CO_2_ER products.

A nitrogen bleed-line approach was used to determine the true gas flow rate reaching the GC (${F}_{{{tot}}}$, see equations below)^55^. In this technique, a known flow rate of N_2_ (${F}_{{{N}}_{2}}$, here we used 20 ml/min) is combined with the gaseous output of the electrolyzer before being fed to the GC for quantitative analysis. The known flow rate of the N_2_ and the measured N_2_ dilution determined by calibrated GC during the experiments are then used for the output flow calculations. Since the measured N_2_ fraction (${X}_{{{N}}_{2}}$) is the ratio of the controlled flow of N_2_ reference gas (${F}_{{{N}}_{2}}$) over the total flow reaching the GC (${F}_{{{tot}}}$, equal to the cell outflow flow plus the N_2_ reference flow rates), i.e. ${X}_{{{N}}_{2}}={F}_{{{N}}_{2}}/{F}_{{{tot}}}$, the unknown *F*_tot_ can simply be determined by this relationship: ${F}_{{{tot}}}={F}_{{{N}}_{2}}/{X}_{{{N}}_{2}}$ and used for subsequently calculating product generation rates.

### Inductively coupled plasma-optical emission spectroscopy (ICP-OES)

The amounts of cations (K^+^) passing through the AEM and GDE cathode were determined by injecting 2.0 ml aliquots of Milli-Q water into the CO_2_ gas inlet flow, letting them pass through the cathode chamber under the pressure of continued CO_2_ flow, and collecting them in a liquid trap downstream of the cell (see scheme in Supplementary Fig. 1c). This approach is not expected to collect all of the K^+^ reaching the cathode, but provides relative information on the cation flux as a function of the varied parameters (anolyte concentration, time). The aliquots were analyzed by ICP-OES (iCAP 7400 Duo MFC ICP-OES analyzer, Thermo Scientific) in axial Ar plasma mode calibrated using K^+^ standards.

### SEM images and EDX analysis

The morphology, elemental mapping and bulk composition of the synthesized catalyst materials were investigated using LEO 1530 Gemini field emission scanning electron microscope (SEM) equipped with a Thermo Scientific UltraDry EDX detector. The SEM images were collected at 4 kV acceleration voltage using a standard aperture size of 30 µm and in-lens secondary electron detector. Elemental mapping (EDX analysis) was acquired at 15 kV acceleration voltage using an aperture size of 60 µm. Survey images at multiple locations of each sample were obtained to assess homogeneity, and the images presented in the manuscript are representative of uniform electrode surfaces. Cross-section samples were prepared via focused ion beam milling using a Zeiss Crossbeam 340 KMAT dual beam instrument with Ga ion source. The milling cut was directly performed without any protection layer using an acceleration voltage of 30 kV and two different polishing currents (80 nA for rough cut and 1.5 nA for fine polishing).

### X-ray photoelectron spectroscopy (XPS)

The surface speciation and composition were studied using a SPECS PHOIBOS 100 analyzer using Al Kα X-ray source (*hν*~1486.74 eV). The XPS spectra were acquired using an energy step of 0.05 eV, dwell time of 0.1 and energy pass of 10 eV with 90 kV and 2200 kV bias and detector voltage, respectively. So-called “quasi in situ” XPS measurements were conducted after the CO_2_ electrolysis experiments were performed under an inert atmosphere inside a glovebox, then the electrodes were removed from the cell, rinsed and dried in the inert environment, and then rapidly transferred from the glovebox to the XPS analysis chamber under vacuum using a specially designed transfer compartment which avoids any air exposure.

The obtained XPS spectra were fitted using CasaXPS software via subtracting a Shirley background. All the spectra were calibrated with respect to the measured adventitious carbon (C 1*s*) peak at 285 eV. The Cu LMM Auger region was collected for the various measuring conditions and fitted using the values for various copper species (Cu_2_O, CuO and Cu) reported by Biesinger^45^, since it is challenging to differentiate between metallic Cu and Cu_2_O species using the Cu 2*p* spectra alone. We also prepared a clean metallic copper reference by Ar sputtering the surface of Cu foil to validate the fitting parameters.

### Operando X-ray absorption spectroscopy (XAS)

The Cu K-edge X-ray absorption measurements were carried out at the KMC-2 beamline at BESSY II, Berlin^56^. The spectra were collected in fluorescence mode using an energy-dispersive detector. XAS was performed in an adapted zero-gap electrochemical reactor with an X-ray transparent window without altering the gas flow pattern and the overall cell geometry to ensure the reproducibility of laboratory results during operando XAS measurements (Supplementary Fig. 9). XANES and EXAFS spectra were collected between 8779 eV and 9729 eV (*K* = 14) for cells at a constant voltage of 3.2 V under varied anolyte conditions. Each EXAFS spectrum required ~40 min and at least three repetitions per sample were collected. Cu K-edge reference spectra for metallic Cu, Cu_2_O, Cu(OH)_2_, basic Cu carbonate and CuO were collected in transmission mode on pellets made from their respective diluted powders with cellulose. All the XAS data were processed via Athena software from Demeter software suite^57^. The metallic Cu spectrum was used as a reference for energy calibration.

## Supplementary information

Supporting information file

Peer review file

## Data Availability

The experimental datasets generated in the current study are available in the Zenodo repository at 10.5281/zenodo.7737136^58^.
