# Supplementary material for: Unintended cation crossover influences CO2 reduction selectivity in Cu-based zero-gap electrolysers
Source: Nat Commun. 2023 Apr 12;14:2062. doi: 10.1038/s41467-023-37520-x (PMC10097803; doi:10.1038/s41467-023-37520-x)
Supplement: Supplementary file 1 — Supporting information file [file 41467_2023_37520_MOESM1_ESM.pdf]

SUPPORTING INFORMATION

# Unintended cation crossover influences $CO_2$ reduction selectivity in Cu-based zero-gap electrolysers

Gumaa A. El-Nagar,[a]* Flora Haun,[a,b] Siddharth Gupta,[a,b] Sasho Stojkovikj,[a,b] Matthew T. Mayer[a]*

[a] Electrochemical Conversion, Helmholtz-Zentrum Berlin für Materialien und Energie GmbH, Hahn-Meitner-Platz 1, D-14109, Berlin, Germany

[b] Institut für Chemie & Biochemie, Freie Universität Berlin, Berlin 14195, Germany

* gumaa.el-nagar@helmholtz-berlin.de, m.mayer@helmholtz-berlin.de

## Supplementary Note 1: Factors determining co-ion concentration in the membrane

Based on Donnan theory and the capacity for ion exclusion by membranes composed of fixed ionic groups, the concentration of co-ions (i.e. solution ions of the same charge as the fixed charges in the membrane) can be estimated based on their concentration in solution and the membrane fixed charge concentration. Helpful explanations and derivations can be found in the following: Barragán et al.,[1] Luo et al.,[2] and Sarkar et al.[3] (for the latter two, see their supporting information files). For reference, we reproduce here the expression presented by Barragán et al.[1] in which the partition coefficient $K$ for the co-ion (defined as the ratio of co-ion concentration in the membrane to that in the external solution $c$) is related to the effective fixed charge density in the membrane $\overline{X}_{eff}$ and the solution concentration $c$:

$$K = -\frac{\overline{X}_{eff}}{2c} + \sqrt{1 + \left(\frac{\overline{X}_{eff}}{2c}\right)^2}$$

This relationship is visualized in the plot below, where $\overline{X}_{eff}$ was set to 2 M (a value approximating the fixed charge density in the PiperION membrane). There it can be seen that for electrolyte concentrations $c$ comparable to the membrane fixed charge density (e.g. $c \geq 1$ M) the co-ion uptake in the membrane is expected to be significant ($K \geq 40\%$), whereas only for $c \ll \overline{X}_{eff}$ are the co-ions strongly excluded.

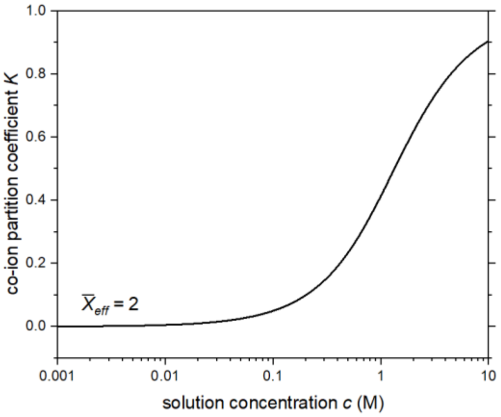

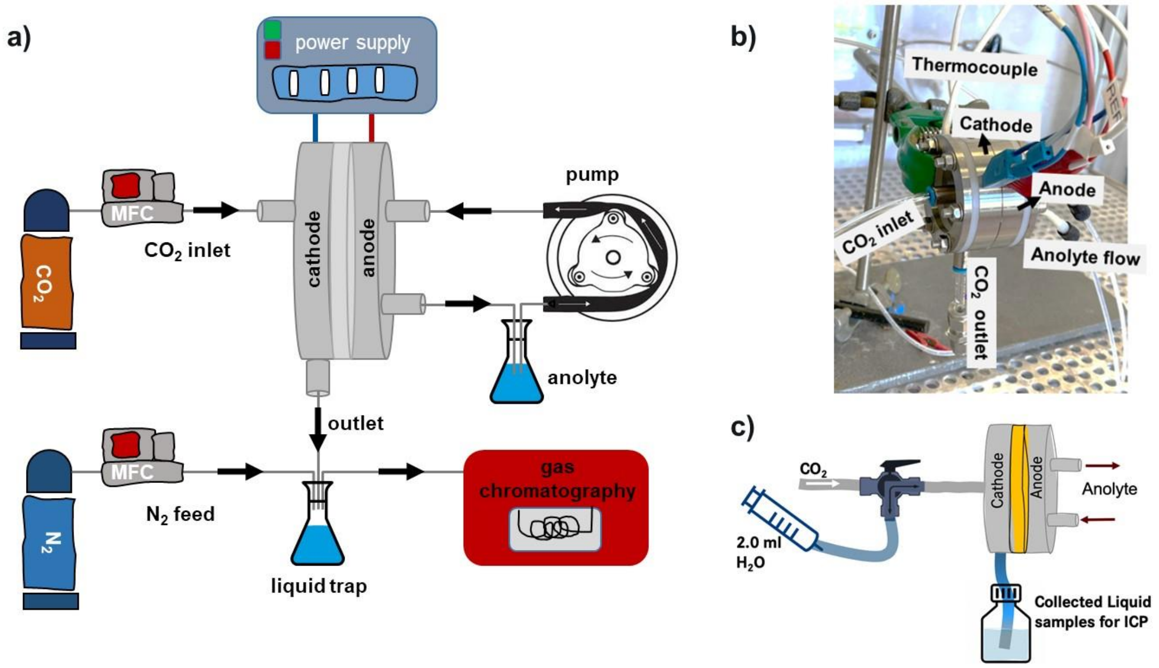


**Figure S1. $CO_2$ electrolyzer experimental setup.** (a) Schematic of electrolyzer test system showing gas and liquid flows. (b) Photo of the assembled cell and connections. (c) Schematic of cathode rinsing method for collecting soluble species formed at the GDE backside for subsequent quantification.

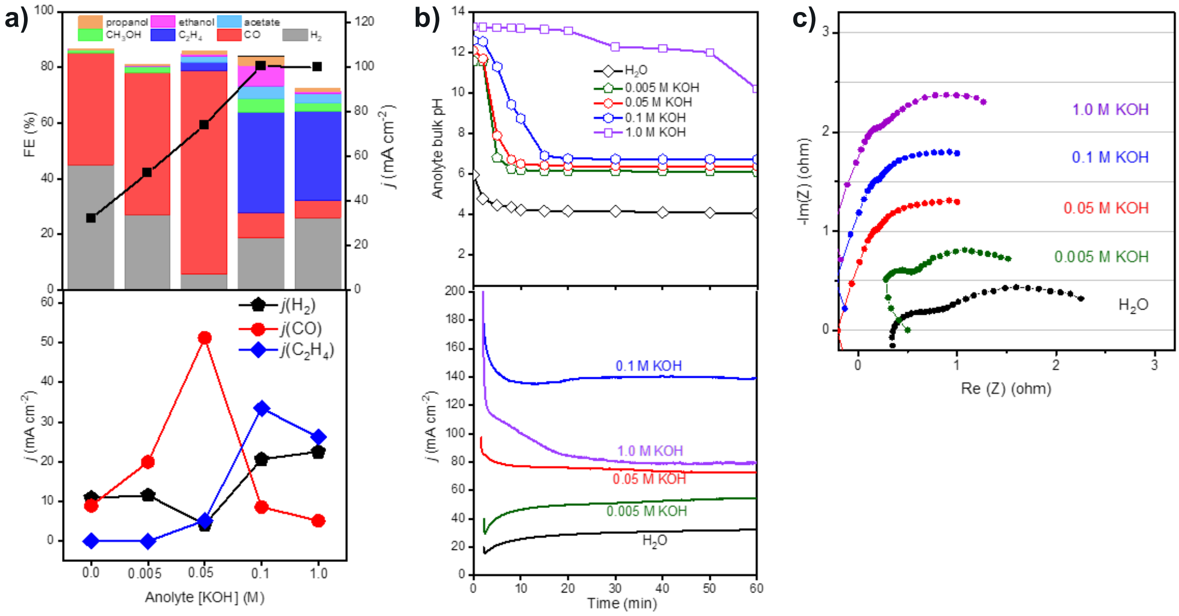


**Figure S2. Dataset for cells operated at 3.2 V with different KOH anolyte concentrations.** (a) Top: product selectivity (FE%, bars) and total current density (*j*, squares) as a function of anolyte KOH concentration (each condition tested using a fresh Cu cathode). Bottom: corresponding partial current densities of the major products. (b) Anolyte pH measurements and total current density over time for cells operated at 3.2 V. (c) Nyquist plots obtained at 2.5 V after $CO_2$ER operation for 2 hours (1.0 Hz-1.0 MHz, 10 mV amplitude; plots are offset vertically for clarity).

For measurements of Fig. 1b conducted at constant cell bias of 3.2 V, corresponding details (pH and total current densities over time) are shown in Fig. S2. In each case, the currents show some initial transient behavior corresponding with the anolyte pH transformation resulting from reaction with $HCO_3^-$ /$CO_3^{2-}$ crossing the membrane,[4,5] but after *ca.* 20 min. they each remain stable over the course of the testing period. Once the pH and current densities stabilize, the devices using [KOH] $\leq$ 0.1 M exhibited stable operation and produced consistent product selectivity over repeated GC injections. At higher concentrations, the current densities were less stable due to precipitation-induced deactivation. Notably, the magnitudes of the current density scale with the anolyte concentration. This could be influenced by differences in ohmic resistance (across the electrolyte and membrane) and by differences in electrode activity resulting from the varied electrolyte concentrations. The measured ohmic resistances were low and rather consistent (0.18–0.22 Ω, Fig. S2c.) despite the wide variation in anolyte ionic strength, suggesting the zero-gap configuration minimizes the sensitivity to electrolyte conductivity.[6] In contrast, the charge transfer resistances do vary, generally decreasing with increased ionic strength. This parameter is sensitive to the kinetics of the electrode processes, suggesting that processes on the anode and/or cathode are influenced by the concentration of cations. In MEA devices, precise deconvolution of the contributions to the 2-electrode cell response is complicated and can be can be aided by numerical modeling, although to our knowledge previous models for MEA $CO_2$ER have not included the effects of anolyte neutralization in alkaline MEA configurations since they assumed an infinite and rapid feed of alkaline anolyte.[6]

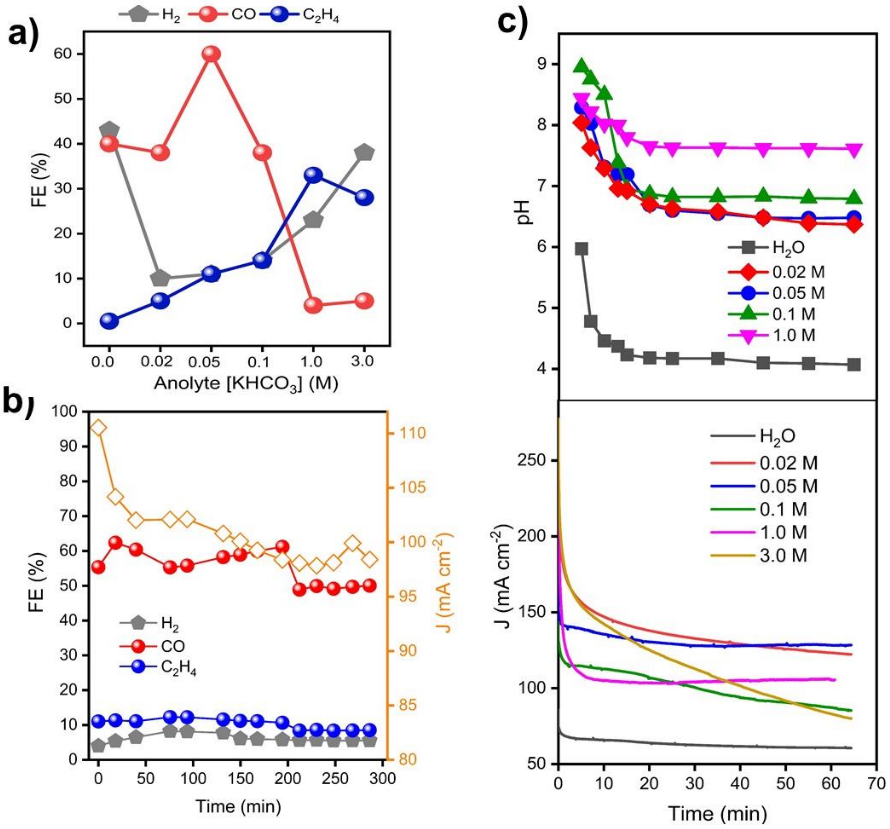


**Figure S3. Cu-MEA $CO_2$ER selectivity using $KHCO_3$ anolytes under constant voltage (3.2 V).** (a) Faradaic efficiency (FE) distribution of the major products $H_2$, $C_2H_4$ and CO as a function of anolyte concentration (x-axis not to scale). Data collected from separate CuNP GDE cathodes tested under each condition. (b) FE and current density vs time for a cell with 0.05 M $KHCO_3$ anolyte operated continuously. All cells used an $IrO_2$ anode and were tested under a two-electrode bias of 3.2 V. (c) Current density and bulk anolyte pH vs time for cells tested using varied [$KHCO_3$] anolytes. Only gaseous major products are shown, while liquids are omitted here for clarity.

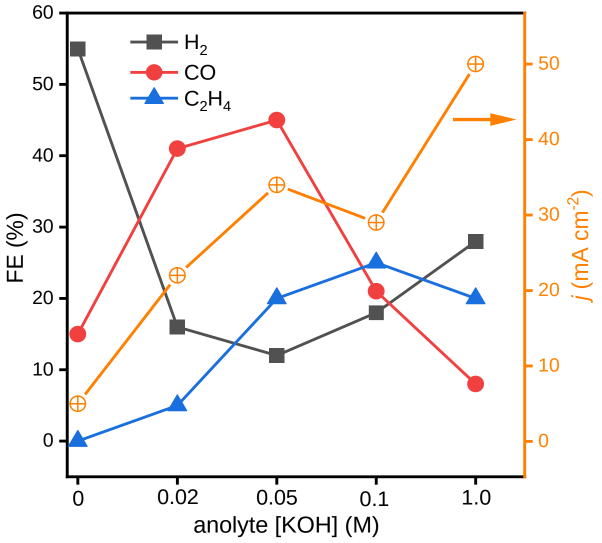


**Figure S4. Cu-MEA cell with Fumasep membrane, selectivity dependence on anolyte concentration.** Faradaic efficiency distribution of the major products generated by Cu-MEA $CO_2$ electrolysis cells using Fumasep FAA-3-pk-75 membranes, operating at constant voltage (3.2 V), as a function of anolyte KOH solution concentration. Orange circles represent the average current density (right y-axis). The x-axis is not to scale, and minor products are omitted for clarity.

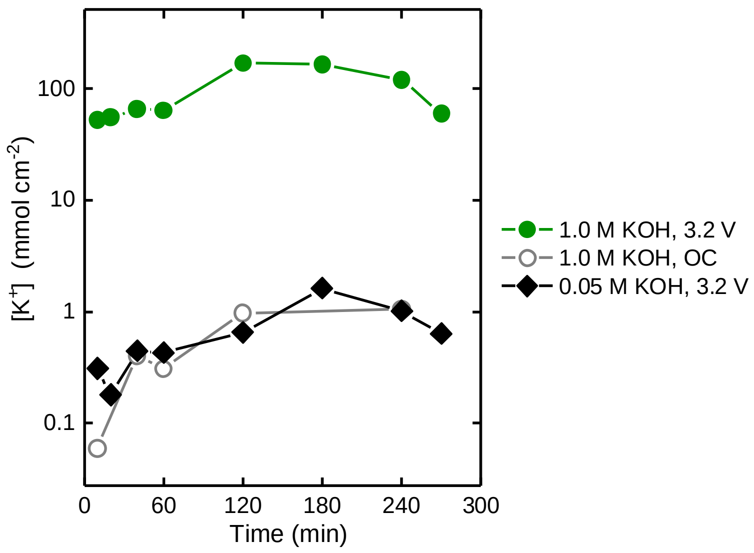


**Figure S5. Magnitude of $K^+$ crossover versus time.** Variation of quantified $K^+$ (collected by flushing the GDE backside with repeated water aliquot injections) over time for zero-gap electrolysers with 1.0 M KOH operated at 3.2 V as well as sitting at open circuit (OC), and 0.05 M KOH operated at 3.2 V. Each point represents the amount collected from a single rinse.

To investigate the combined effects of concentration and electrochemistry on the magnitude of $K^+$ crossover, we conducted a series of tests where the cathode was rinsed repeatedly to sample $K^+$ over an extended period. The cation quantification is shown in Fig. S5. Note that since the act of repeated rinsing changes the amount of cations at the cathode, this data is used for qualitative observation of trends only. First, comparing cells operating under $CO_2$ER conditions with concentrated and dilute anolytes, we see that even in the first minutes, the 1 M KOH leads to $K^+$ levels more than two orders of magnitude greater than the 0.05 M case. During extended operation, the concentrated anolyte gives consistently high values but with irregular behavior, likely due to the formation of significant solid precipitates throughout the GDE which are incompletely collected by each rinse. The dilute anolyte also showed some increase in $K^+$ crossover with time, but never reached high values. For comparison, a cell with 1 M KOH was assembled and allowed to simply sit at open circuit, repeating the cathode rinsing procedure. In this case, the $K^+$ crossover amount remained small, far below that of the same configuration under $CO_2$ER conditions. This suggests that electrochemical conditions also play an important role in activating $K^+$ crossover. This could arise in part from modulation of the interfacial Donnan potential (at the membrane-solution interface), but also due to phenomena such as electrowetting.

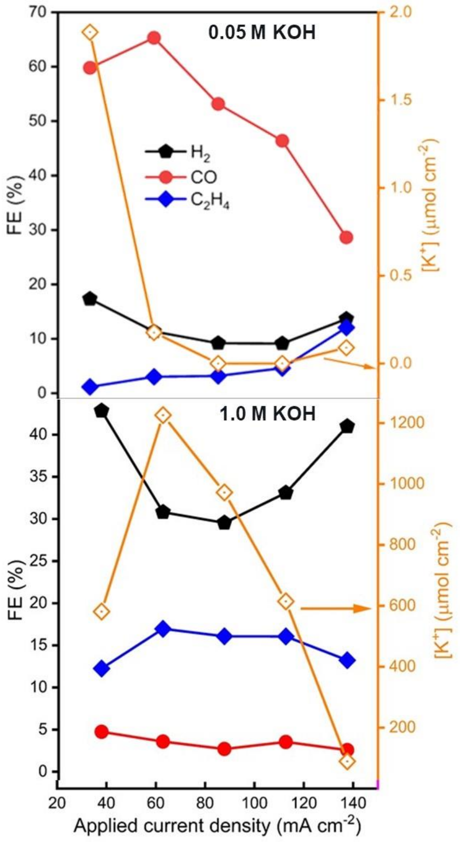


**Figure S6. FE from controlled current experiments (from dataset of Fig. 1c)**. Identical cells were assembled and operated with anolytes of (a) 0.05 M or (b) 1.0 M KOH. For each anolyte, the data represent the behavior of a single cell operated under fixed applied current densities stepping from low to high, holding 50 min at each current, and performing several GC injections at each. After each current step, the cathode rinse protocol was applied and the measured $K^+$ is shown (right y-axis). Due to the nature of the experiment (single electrodes run at range of currents), liquid products were not quantified. Minor gaseous products are also omitted for clarity.

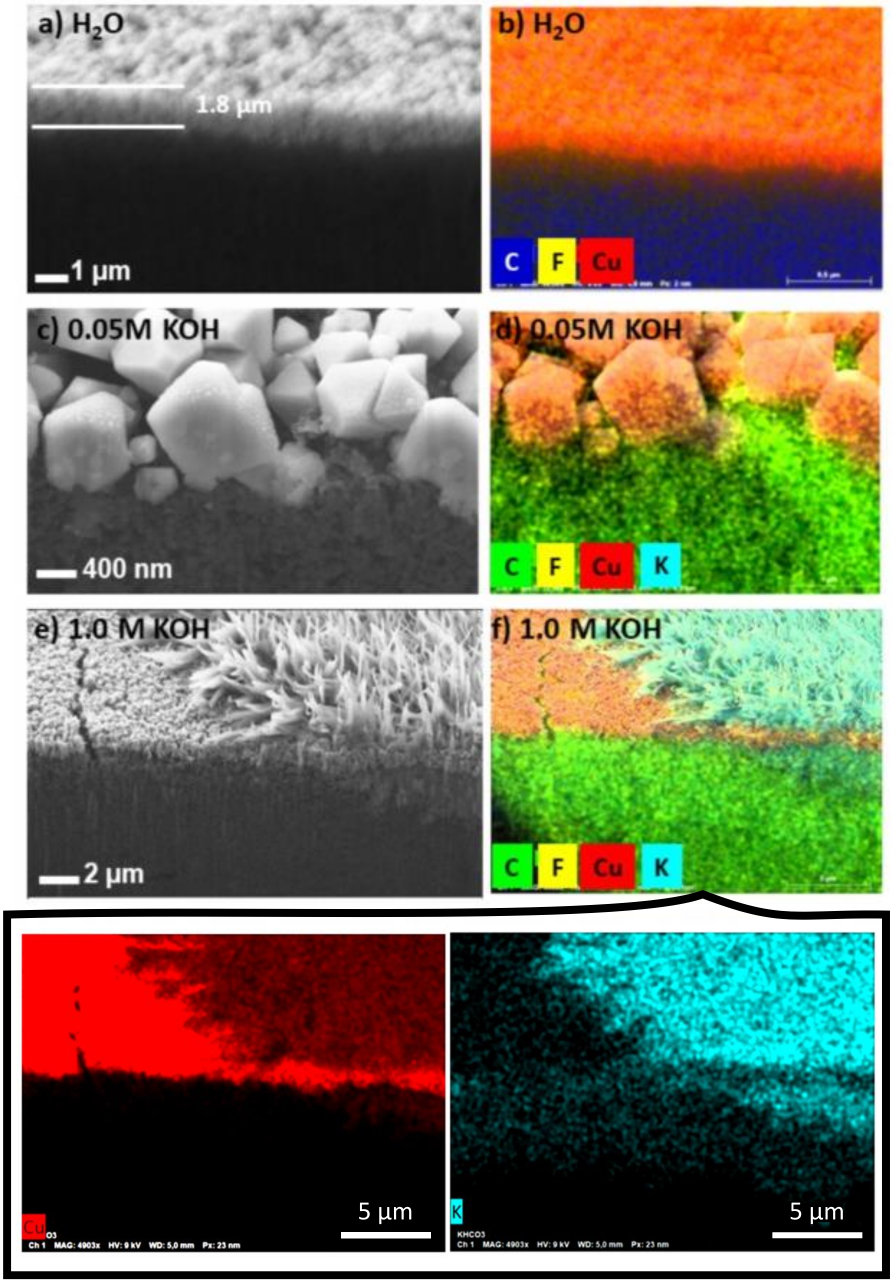


**Figure S7. Catalyst layer structure analysis after operation using various anolytes.** SEM images at FIB cross sections (a, c, e) and respective EDX elemental mapping (b, d, f) of Cu-coated GDE cathodes after 4.5 h $CO_2$ER testing at 3.2 V using anolytes of $H_2O$, 0.05 M KOH, and 1 M KOH (as indicated). The carbon component is the microporous layer of the GDE. The bottom inset shows individual maps of Cu and K from figure (f).

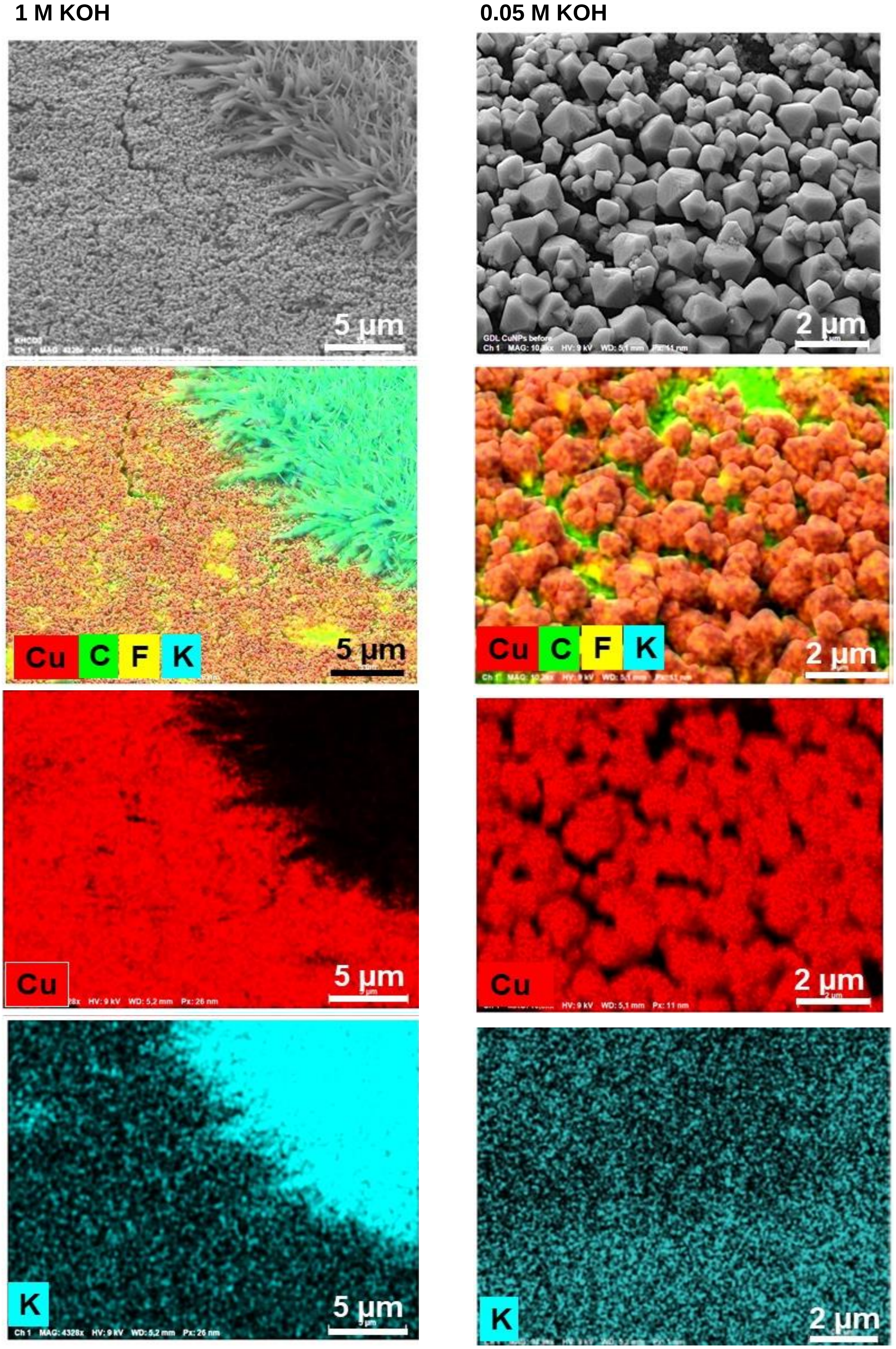


**Figure S8**. **Catalyst layer structure analysis after operation using various anolytes.** Top-view SEM and EDX elemental mapping of gas diffusion electrode catalyst surface after operation in zero-gap electrolyzer using 1.0 M (left) and 0.05 M (right) KOH anolytes.

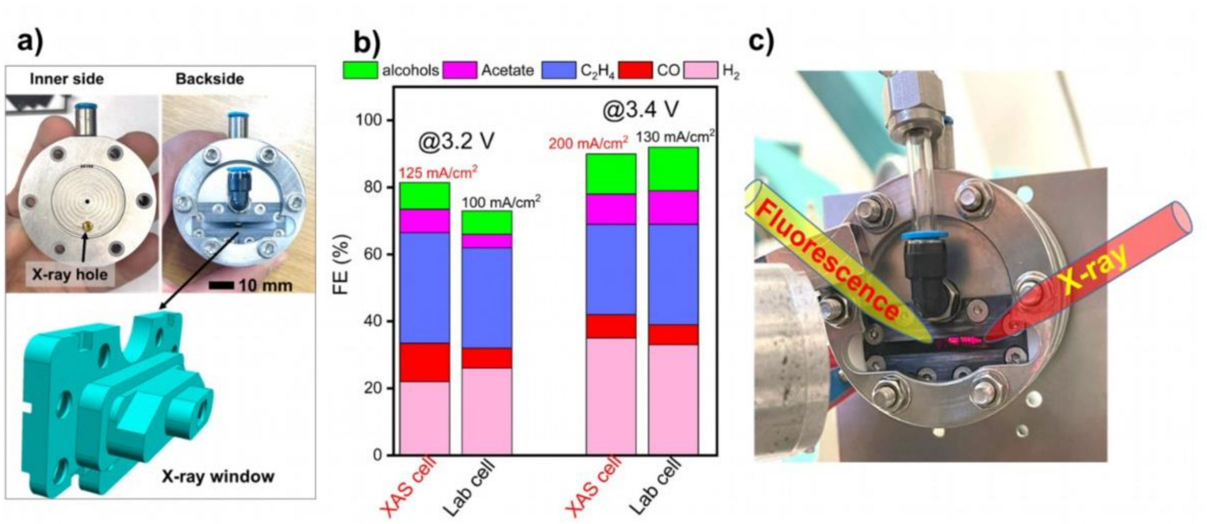


**Figure S9. X-ray absorption spectroscopy cell.** (a) Photos and 3D rendering showing the operando XAS zero-gap electrochemical reactor adapted with an X-ray transparent window. (b) comparing the FEs and current density of the modified cell (XAS cell) and the normal lab cell. (c) photo of in-situ XAS during in-situ XAS measurements at beamline KMC-2 at BESSY II.

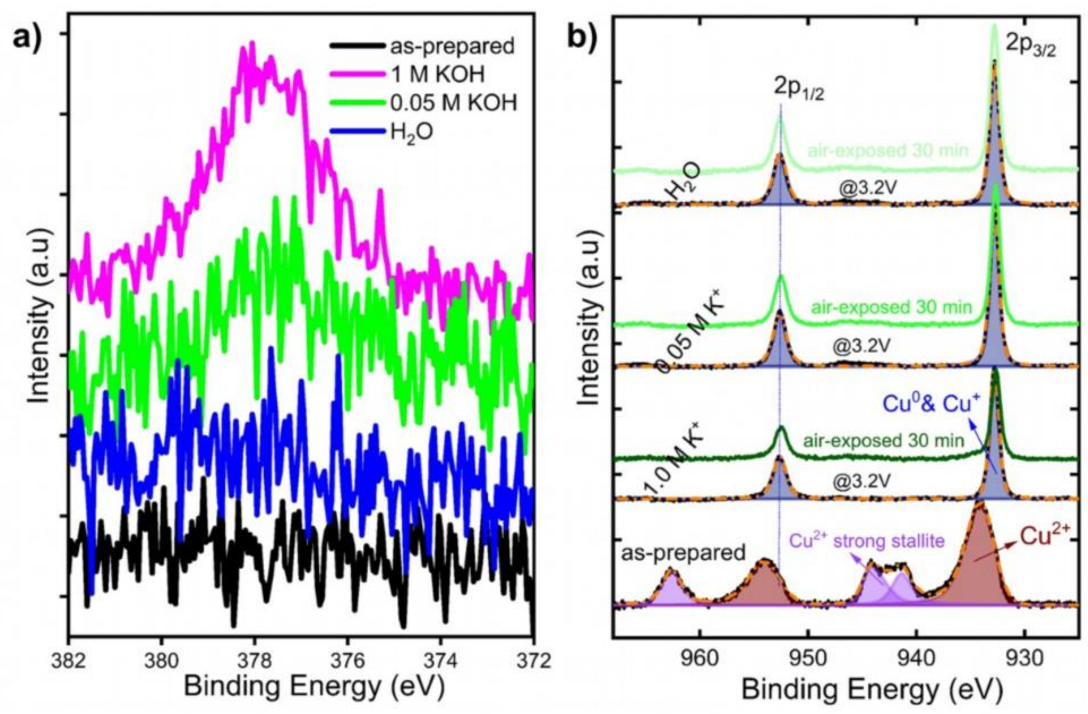


**Figure S10. XPS analysis detail.** (a) XPS spectra of K 2s of the cathodes operated with pure $H_2O$ and various KOH anolyte concentrations (0.05 M and 1.0 M). (b) XPS spectra of Cu 2p for the as-prepared Cu cathodes and Cu cathodes operated with pure $H_2O$, 0.05 M KOH and 1.0 M KOH at 3.2 V for 1.0 hours. The green plots showed their respective Cu 2p spectra after brief air exposure (30 minutes).

## References


1 Barragán, V. M. & Pérez-Haro, M. J. Correlations between water uptake and effective fixed charge concentration at high univalent electrolyte concentrations in sulfonated polymer cation-exchange membranes with different morphology. *Electrochim Acta* **56**, 8630-8637 (2011). https://doi.org/10.1016/j.electacta.2011.07.060

2 Luo, S., White, W., Cardon, J. M. & Ardo, S. Clarification of mechanisms of protonic photovoltaic action initiated by photoexcitation of strong photoacids covalently bound to hydrated Nafion cation-exchange membranes wetted by aqueous electrolytes. *Energ Environ Sci* **14**, 4961-4978 (2021). https://doi.org/10.1039/d1ee00482d

3 Sarkar, S., SenGupta, A. K. & Prakash, P. The Donnan membrane principle: opportunities for sustainable engineered processes and materials. *Environ Sci Technol* **44**, 1161-1166 (2010). https://doi.org/10.1021/es9024029

4 Ma, M. *et al.* Insights into the carbon balance for CO2 electroreduction on Cu using gas diffusion electrode reactor designs. *Energ Environ Sci* **13**, 977-985 (2020). https://doi.org/10.1039/d0ee00047g

5 Vass, Á. *et al.* Local Chemical Environment Governs Anode Processes in CO2 Electrolyzers. *ACS Energy Letters* **6**, 3801-3808 (2021). https://doi.org/10.1021/acsenergylett.1c01937

6 Weng, L.-C., Bell, A. T. & Weber, A. Z. Towards membrane-electrode assembly systems for CO2 reduction: a modeling study. *Energ Environ Sci* **12**, 1950-1968 (2019). https://doi.org/10.1039/c9ee00909d
